# Supplementary figures and images for: Ultrafast high-harmonic nanoscopy of magnetization dynamics
Source: Nat Commun. 2021 Nov 3;12:6337. doi: 10.1038/s41467-021-26594-0 (PMC8566501; doi:10.1038/s41467-021-26594-0)

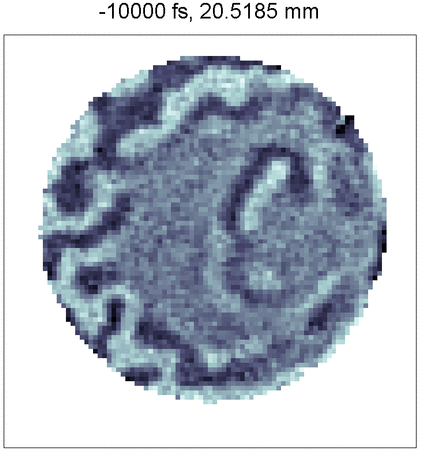

Supplement: Supplementary file 1 — Supplementary Movie 1 [file 41467_2021_26594_MOESM1_ESM.gif]

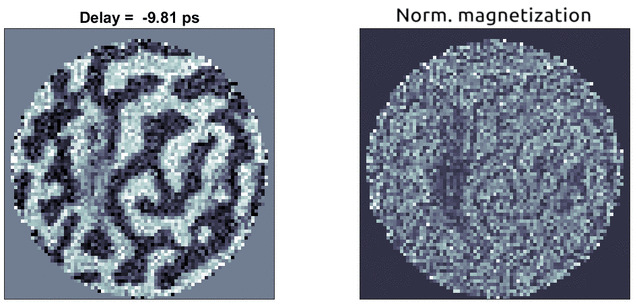

Supplement: Supplementary file 2 — Supplementary Movie 2 [file 41467_2021_26594_MOESM2_ESM.gif]

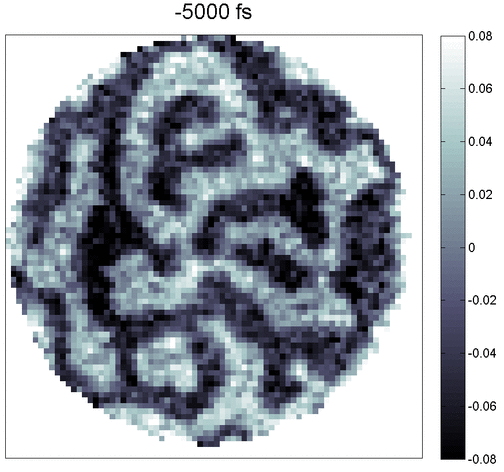

Supplement: Supplementary file 3 — Supplementary Movie 3 [file 41467_2021_26594_MOESM3_ESM.gif]

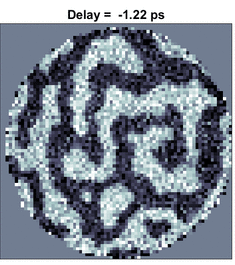

Supplement: Supplementary file 4 — Supplementary Movie 4 [file 41467_2021_26594_MOESM4_ESM.gif]

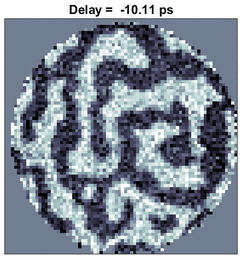

Supplement: Supplementary file 5 — Supplementary Movie 5 [file 41467_2021_26594_MOESM5_ESM.gif]
