# Supplementary material for: Ultrafast high-harmonic nanoscopy of magnetization dynamics
Source: Nat Commun. 2021 Nov 3;12:6337. doi: 10.1038/s41467-021-26594-0 (PMC8566501; doi:10.1038/s41467-021-26594-0)
Supplement: Supplementary file 7 — Description of Additional Supplementary Files [file 41467_2021_26594_MOESM7_ESM.pdf]

Title: Supplementary Movie 1

Description: Ultrafast demagnetization movie using high pump fluence.

Title: Supplementary Movie 2

Description: Ultrafast demagnetization movie using 1.4(1) mJ/cm2 pump fluence. Marked as Fluence 3 in Fig. 5.

Title: Supplementary Movie 3

Description: Ultrafast demagnetization movie using 1.2(2) mJ/cm2 pump fluence. Marked as Fluence 1 in Fig. 4.

Title: Supplementary Movie 4

Description: Ultrafast demagnetization movie using 1.3(1) mJ/cm2 pump fluence. Marked as Fluence 2 in Fig. 4.

Title: Supplementary Movie 5

Description: Ultrafast demagnetization movie using 1.3(1) mJ/cm2 pump fluence. The data is recorded after Supplementary Dataset 4 for verification.
